# Neurocysticercosis and developmental venous anomaly in close proximity: a case report

**DOI:** 10.11604/pamj.2025.52.19.46826

**Published:** 2025-09-16

**Authors:** Dirkina Jacoba Wessels, Jerry George, Keitumetse Manoko, Sujay Surendran-Nair, Thendo Nemutudi, Andre Mochan

**Affiliations:** 1 Division of Neurology, Department of Neurosciences, School of Clinical Medicine, Faculty of Health Sciences, University of the Witwatersrand, Johannesburg, South Africa

**Keywords:** Neurocysticercosis, developmental venous anomaly, epilepsy, case report

## Abstract

Neurocysticercosis (NCC) is a leading cause of acquired epilepsy and other neurological disturbances worldwide. Developmental venous anomaly (DVA) is the most common cerebral vascular malformation, typically asymptomatic and discovered incidentally. This case report illustrates the rare instance of a DVA found adjacent to an NCC lesion on imaging. The patient is a 26-year-old male presenting with new-onset seizures. The diagnosis was made on MRI brain and serum cysticercosis antibody testing. Limited literature is available on the coexistence of these two entities, prompting contemplation about the implications for diagnosis, management, and prognosis.

## Introduction

Neurocysticercosis (NCC), caused by the larval form of the tapeworm *Taenia solium*, is a leading cause of acquired epilepsy and other neurological disturbances worldwide, particularly in regions with poor sanitation. The clinical presentation of NCC is diverse, ranging from asymptomatic to severe neurological disease, depending on the number, size, stage, and location of the cysts, as well as the host immune response [1].

Developmental venous anomalies (DVAs) are the most common cerebral vascular malformation, typically asymptomatic and often discovered incidentally during neuroimaging for unrelated reasons [2]. DVAs are congenitally irregularly arranged small veins that drain circularly into a larger central vein. They are considered benign and clinically silent. However, their presence may become relevant when associated with other pathologies, such as NCC, where the associated DVA might influence disease manifestations or complicate management.

## Patient and observation

**Patient information:** a 26-year-old male patient, first-degree relative (twin sister) with epilepsy, no significant medical or trauma history.

**Clinical findings:** the patient was admitted with right-sided focal motor seizures with secondary generalisation. He had had a total of three episodes since the onset eight months prior to presentation. His clinical examination was normal with no focal neurological signs.

**Timeline of current episode:** August 2022: presentation to hospital, blood tests (full blood count, inflammatory markers, urea and electrolytes, calcium, magnesium, and phosphate), and an electroencephalogram was done. October 2022: computed tomography (CT) of the brain was done. April 2023: magnetic resonance imaging (MRI) of the brain was done.

**Figure 1:**
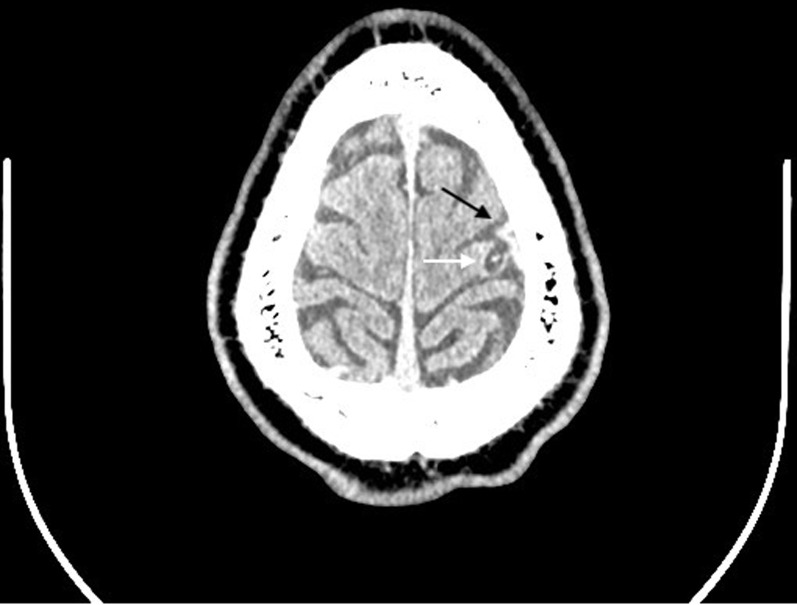
contrast-enhanced axial computed tomography brain showing a developmental venous anomaly (black arrow) adjacent to a cystic lesion in the posterior frontal lobe without surrounding oedema (white arrow)

**Figure 2:**
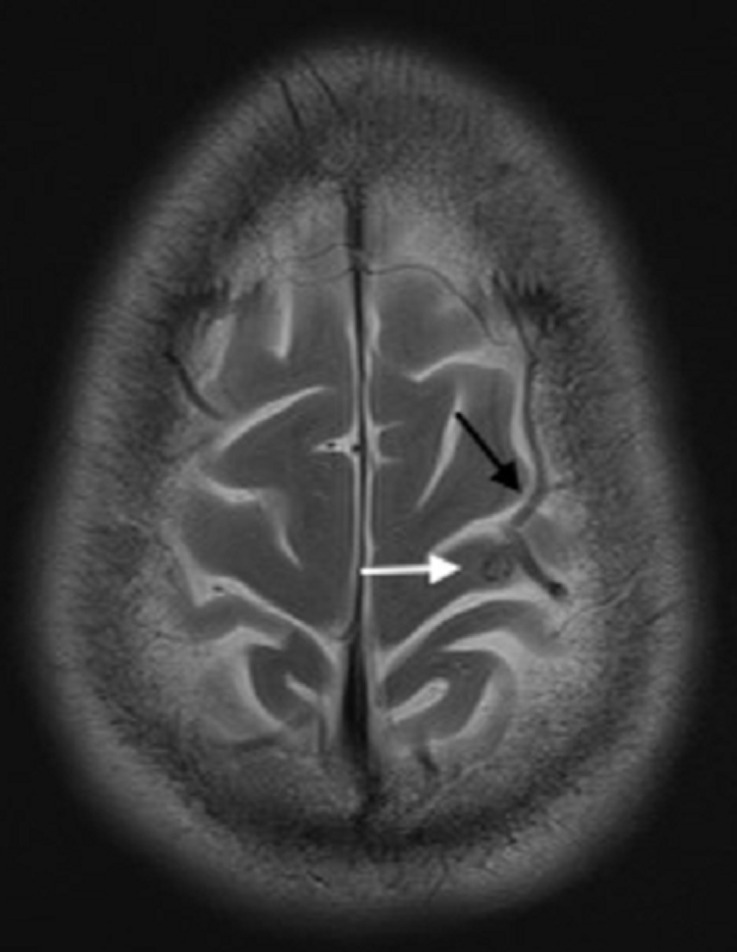
axial T2-weighted magnetic resonance imaging brain showing a non-thrombosed developmental venous anomaly in the left frontal region (black arrow) adjacent to a cystic lesion typical of vesicular-stage neurocysticercosis (white arrow)

**Diagnostic assessment:** blood tests (full blood count, inflammatory markers, urea and electrolytes, calcium, magnesium, and phosphate) and electroencephalogram were unremarkable. CT brain showed a prominent high left frontal vein with intraparenchymal extension into the left lateral ventricle, suggestive of a developmental venous anomaly (DVA), and an adjacent cystic lesion in the pre-central gyrus of the posterior frontal lobe with no surrounding oedema (Figure 1). Subsequent MRI brain showed the prominent left frontal central vein, traversing white and grey matter, draining into the superior sagittal sinus and entering the lateral ventricle wall, indicative of non-thrombosed DVA. The adjacent cystic lesion was typical of the vesicular stage of NCC (Figure 2). Following this, NCC serology was performed, which confirmed cysticercosis IgG antibodies.

**Diagnosis:** the results were consistent with a DVA with an adjacent NCC lesion, vesicular stage.

**Therapeutic interventions:** prednisone 1mg/kg/ day for 5 days followed by albendazole 400mg orally b.d. (15mg/kg/day) for 2 weeks. Epilim 500mg orally b.d.

**Follow-up and outcome of interventions:** subsequently he had no further seizures.

**Patient perspective:** *”I understand that I am diagnosed with epilepsy and will now take medication for the rest of my life. Since I started the treatment, I have not had any more seizures”*.

**Informed consent:** written informed consent was obtained from the patient for publication of this case.

## Discussion

**Figure 3:**
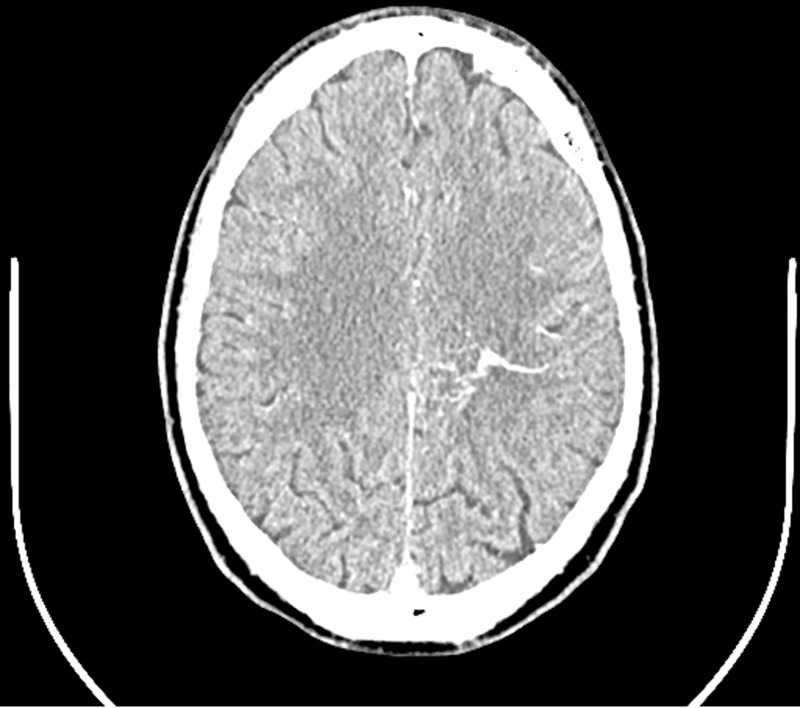
post-contrast axial computed tomography brain demonstrating the characteristic “caput medusae” sign of a developmental venous anomaly, formed by multiple small medullary veins converging into a larger collector vein

Developmental venous anomalies are vascular malformations. There are variations in the medullary veins that drain the grey and white matter [3]. A DVA comprises a network of thin-walled medullary veins draining into a collector vein, which then empties into either a superficial or deep cerebral vein. This network of veins feeding into a collector vein resembles ‘medusa´s head´ or caput medusae (Figure 3). The cause or origin of DVAs is not well understood, but according to current theory, there is aplasia, hypoplasia or early occlusion of developing medullary veins. As a result of the maldevelopment, there will be compensatory development of the DVA due to the absence of normal medullary veins [2].

Developmental venous anomalies (DVAs) are generally clinically asymptomatic, being typically discovered incidentally on imaging. The reported clinical spectrum of DVA presentations based on a systematic review dating from 2009 is 61% asymptomatic, 23% non-specific clinical features, 6% with focal deficit, 4% seizures and less than 1% with infarction [4]. In symptomatic DVAs, the presentation is either due to mechanical obstruction, flow-related or idiopathic. Regarding mechanical obstruction, the DVA can lead to hydrocephalus or compression of neurovascular structures, causing symptoms. Flow-related symptomatic presentation can be due to high flow (micro-shunt into DVA, or arteriovenous malformation) or low flow (venous obstruction or thrombosis) [3].

The imaging modality of choice for visualising DVA is MRI brain [5]. Treatment of DVA if asymptomatic is not indicated because of the complex venous drainage and the risk of a subsequent venous infarction [2]. If the DVA becomes thrombosed, however, then the patient should receive anticoagulation and be investigated for pro-thrombotic states; if the DVA develops haemorrhage the patient may need surgical management [2,5].

Neurocysticercosis is the commonest helminthic disease of the central nervous system. It is caused by the adult *Taenia solium* helminth. Transmission is through the faecal-oral route. The major risk factors for acquiring the disease are low socio-economic status, poor sanitation and water scarcity, all contributing to poor hand hygiene [6]. The prevalence of NCC in South Africa is 10-20%, with pig farming areas having the highest prevalence [7].

There is no known relationship between DVA and NCC. NCC can present with a variety of clinical syndromes according to the size, number and location of lesions. With parenchymal NCC, seizures and headaches are the commonest clinical manifestations. Acquired epilepsy accounts for up to 70% of all reported cases of NCC, while headaches account for up to 30% [6,8]. A subset of patients presents with extraparenchymal NCC characterised by involvement of the meningeal, subarachnoid, and/or intraventricular spaces [6,9]. In the meningeal and subarachnoid form, called racemose cysticercosis, presentations include meningitis, raised intracranial pressure, and cranial neuropathies [9]. With intraventricular NCC, the clinical syndromes of hydrocephalus and intracranial hypertension are typically observed; these occur secondary to obstruction of cerebrospinal fluid pathways by cysticerci [6,8,9]. Both parenchymal and extraparenchymal NCC can additionally present with focal neurological deficits like hemiparesis, brainstem syndromes and visual field defects [6,9]. These occur either secondary to compression of the adjacent brain tissue by lesions or infarction of brain tissue as a consequence of NCC-induced arteritis [6,9]. In its most severe form, NCC may present as cysticercosis encephalitis; this occurs with a large burden of cysticerci, inducing an overwhelming immune response [6]. Parenchymal and extraparenchymal NCC may co-occur in the same patient.

Serum NCC antibody testing and neuroimaging form the basis of diagnosis for NCC. A positive serum cysticercosis IgM antibody is indicative of recent infection, while a positive IgG antibody is indicative of previous infection.

The radiological changes are dependent on the stage of development of the cysticerci. There are four recognised stages: vesicular cysticerci are small, rounded cysts with an internal hyperdense nodule representing the scolex, giving the pathognomonic “hole-with-dot” appearance diagnostic of NCC [10]. The colloidal and nodular-granular cysticerci appear as ill-defined lesions with associated perilesional oedema and a ring or a nodular pattern of enhancement post-contrast administration [6]. Finally, calcified cysticerci appear as small hyperdense nodules with no associated perilesional oedema or abnormal enhancement post-contrast administration [6].

Neurocysticercosis (NCC)-specific treatment comprises administration of cysticidal drugs, namely praziquantel and albendazole. These are either used individually or in combination as guided by the stage, number and site of the cysticerci as well as drug availability [6].

Our patient presented with seizures. Initial CT scan of the brain showed a DVA with features suggestive of either an outpouching of the DVA or a thrombosis (Figure 1). MRI brain then revealed the queried outpouching/thrombosed DVA to be a vesicular stage NCC lesion adjacent to the DVA mimicking an outpouching/thrombosis (Figure 2).

## Conclusion

This patient with new-onset seizures presents a rare and intriguing instance where a lesion of vesicular NCC was incidentally identified adjacent to a DVA on imaging studies. The co-occurrence of these two distinct entities in proximity poses unique diagnostic and therapeutic challenges. It prompts contemplation about the potential interactions between a parasitic infection and a vascular malformation in the brain, exploring whether the presence of a DVA could impact the local inflammatory response to NCC or vice versa. This curious close co-occurrence of DVA and NCC cyst has not been described before.
